# Comparison of a low carbohydrate and low fat diet for weight maintenance in overweight or obese adults enrolled in a clinical weight management program

**DOI:** 10.1186/1475-2891-6-36

**Published:** 2007-11-01

**Authors:** James D LeCheminant, Cheryl A Gibson, Debra K Sullivan, Sandra Hall, Rik Washburn, Mary C Vernon, Chelsea Curry, Elizabeth Stewart, Eric C Westman, Joseph E Donnelly

**Affiliations:** 1 Department of Kinesiology and Health Education, Southern Illinois University Edwardsville, Edwardsville, USA; 2 General and Geriatric Medicine, University of Kansas Medical Center, Kansas City, USA; 3 Dietetics and Nutrition, University of Kansas Medical Center, Kansas City, USA; 4 University of Kansas Medical Center, Kansas City, USA; 5 Center for Physical Activity and Weight Management, University of Kansas, Lawrence, USA; 6 Private Bariatric and Family Practice, Lawrence, USA; 7 Vince & Associates Clinical Research, Overland Park, USA; 8 TransforMED, Leawood, KS, USA; 9 Department of Medicine, Duke University, Durham, USA

## Abstract

**Background:**

Recent evidence suggests that a low carbohydrate (LC) diet may be equally or more effective for short-term weight loss than a traditional low fat (LF) diet; however, less is known about how they compare for weight maintenance. The purpose of this study was to compare body weight (BW) for participants in a clinical weight management program, consuming a LC or LF weight maintenance diet for 6 months following weight loss.

**Methods:**

Fifty-five (29 low carbohydrate diet; 26 low fat diet) overweight/obese middle-aged adults completed a 9 month weight management program that included instruction for behavior, physical activity (PA), and nutrition. For 3 months all participants consumed an identical liquid diet (2177 kJ/day) followed by 1 month of re-feeding with solid foods either low in carbohydrate or low in fat. For the remaining 5 months, participants were prescribed a meal plan low in dietary carbohydrate (~20%) or fat (~30%). BW and carbohydrate or fat grams were collected at each group meeting. Energy and macronutrient intake were assessed at baseline, 3, 6, and 9 months.

**Results:**

The LC group increased BW from 89.2 ± 14.4 kg at 3 months to 89.3 ± 16.1 kg at 9 months (*P *= 0.84). The LF group decreased BW from 86.3 ± 12.0 kg at 3 months to 86.0 ± 14.0 kg at 9 months (*P *= 0.96). BW was not different between groups during weight maintenance (*P *= 0.87). Fifty-five percent (16/29) and 50% (13/26) of participants for the LC and LF groups, respectively, continued to decrease their body weight during weight maintenance.

**Conclusion:**

Following a 3 month liquid diet, the LC and LF diet groups were equally effective for BW maintenance over 6 months; however, there was significant variation in weight change within each group.

## Background

Multiple treatment strategies are available for weight loss including energy restriction, physical activity, and/or behavioral modification. However, as noted by Wing and Phelan, only 20% of overweight individuals losing weight are successful for weight maintenance when defined as losing at least 10% of initial body weight and maintaining the loss for at least 1 year [1,2]. Thus, improved strategies to prevent weight re-gain are needed.

Recently, diets lower in carbohydrate and higher in protein have shown promise for weight loss when compared to typical reduced energy and fat diets. In particular, multiple studies indicate that a low carbohydrate diet may produce greater weight loss than a traditional low fat diet over 6 months and may be comparable to a low fat diet over 12 months [3-7]. Despite the evidence supporting a low carbohydrate diet as an effective tool for weight loss its effect for weight maintenance is unclear. Therefore, the purpose of this study was to compare body weight re-gain in overweight and obese adults consuming a low carbohydrate or traditional low fat diet over 6 months of weight maintenance subsequent to 3 months of weight loss.

## Methods

### Participants

This study was approved by the Human Subjects Committee at the University of Kansas and participants provided informed consent prior to participation in the study. Participants were recruited through advertisements, fliers, and word of mouth. Participants were healthy adults, 19 to 70 years of age, previously sedentary, and overweight or obese (BMI > 27 kg/m^2^). Individuals were excluded if they smoked, used special diets (i.e. vegetarian), were unable to exercise (i.e. walk), were pregnant or lactating, or were in active counseling for any psychological or psychiatric condition. Prior to participation, a physician evaluated each individual to determine potential health risks relative to participation in the study. Individuals were excluded for any metabolic disease affecting energy balance (e.g. diabetes mellitus, cancer, etc.). Except for the exclusion criteria stated above, there were no restrictions for gender, race, or socioeconomic status.

### Study design

This study was conducted in the context of a weight management clinic. All participants received 3 months of a weight loss diet followed by 6 months of a weight maintenance diet either low in carbohydrate or fat. A quazi-experimental design was utilized where clinic site was assigned as either low carbohydrate or low fat; however, analysis was per participant. A total of six separate cohorts (~15–20 participants each) were recruited; 3 low carbohydrate and 3 low fat. Cohorts within each dietary intervention did not differ by protocol, format, or instruction.

### Weight management clinics

Weight management clinic meetings were approximately 90 min and were held weekly for the first 6 months and biweekly for the subsequent 3 months. Clinics were conducted in a group format of 15–20 individuals and each meeting began with a check-in to ensure adherence to the protocol of the study. During check-in, all participants were weighed and provided their self-reported weekly data including: # of liquid shakes consumed (weight loss period only), total g of carbohydrate or fat (weight maintenance period only), min of physical activity (PA), and number of steps recorded by step counters. Following check-in, a 30–45 min presentation was given including instruction in behavioral lifestyle modification, exercise, or nutrition.

In order to increase accountability and protocol compliance participants were asked to provide a mid-week check-in via phone, fax, or email during the first 6 months of the study. For the mid-week check-in participants provided their weekly data (PA, steps, etc.) and presented any concerns related to the study they might have had since the previous meeting. When group meetings changed to bi-weekly, check-ins occurred during the week groups did not meet. All group meetings were lead by the same staff of registered dieticians, exercise physiologists, and behavioral therapists using an identical, standardized protocol.

The only difference in group meetings occurred when the meeting topic was nutrition. All participants consuming a low carbohydrate diet received information and strategies for achieving a diet low in carbohydrate such as shopping, cooking, label reading, etc., and the participants consuming a low fat diet received information and strategies for eating a diet low in fat. Attendance was expected at group meetings. Prior to participation in the study, participants agreed to comply with a 75% attendance rate requirement and understood that they would be terminated from further participation in group meetings if their attendance fell below 75%.

### Very low-energy diet

Weight loss was facilitated using a very low-energy diet (VLED) comprised of 2177 kJ/day for 3 months. During VLED, we utilized a milk-based product (Health Management Resources, Boston, MA) consumed primarily as a liquid shake at 5 intervals throughout the day. Each liquid shake included approximately 435 kJ, 13–17 g of carbohydrate, 1 g of fat, 10–14 g of protein. In addition, a vitamin and mineral supplement was taken twice daily. If participants did not lose at least 10% of their initial body weight during VLED, they were not allowed to continue with the study. The liquid meal replacements were the only source of nutrition during VLED with the exception of non-caloric beverages that were consumed ad libitum. To ensure compliance to the VLED, participants reported their total number of liquid shakes consumed for the previous week at each group meeting.

### Weight maintenance diet

During month 4, a progressive re-feeding schedule was utilized that decreased the number of liquid shakes and increased the number of solid foods consumed each week. This was done to limit adverse events (e.g., nausea, diarrhea, etc.) associated with the transition from the liquid to the solid food diet. Further, the low carbohydrate group was re-fed with solid foods that were low in carbohydrate, such as green leafy vegetables, broccoli florets, lean meats, and nuts and the low fat group was re-fed with low fat foods, such as fruits, vegetables, potatoes, and whole grains.

At the end of month 4, all participants were provided a gram level of carbohydrate or fat based upon their weight maintenance energy requirements. For the low carbohydrate group, the upper limit of carbohydrate grams to be consumed each day was ~20% of their total maintenance energy level and for the low fat group the upper limit of fat grams to be consumed each day was ~30% of their total maintenance energy level. Maintenance energy intakes were calculated using the Harris-Benedict equation to estimate resting energy expenditure (REE) and we used 1.4 × REE to adjust for PA levels [8].

To monitor adherence to the diet, the low carbohydrate group kept a daily tally of grams of carbohydrate consumed and the low fat group kept a daily tally of the number of fat grams consumed, based upon the percentages previously listed, and reported their daily gram total at each weekly meeting. To increase the likelihood that participants would eat according to their diet, group meetings emphasized food label reading, low carbohydrate or low fat food preparation, low carbohydrate or low fat food items and low carbohydrate or low fat food recipes, etc. If participants tended to exceed their allotted number of daily carbohydrate or fat grams, a member of the research staff provided dietary counseling to the particular participant.

### Body weight and regional adiposity

Weights were obtained at the beginning of each group meeting using a digital scale (Befour, Inc., Saukville, WI) accurate to ± 0.1 kg with participants wearing normal clothing without shoes. To calculate BMI, height was measured at baseline using a stadiometer (Perspective Enterprises, Portage, MI). Body Mass Index was calculated as weight in kg divided by height in meters squared (kg/m^2^). Waist circumference was measured at the narrowest portion of the abdomen and hip circumference was measured at the widest portion of the buttocks [9]. Waist and hip circumference were assessed at baseline, 3, 6, and 9 months by obtaining 2 measurements per site within 2 cm using a spring-loaded tape measure (Creative Health Products, Ann Arbor, MI).

### Energy intake

In order to determine compliance to the diet, 3-day food records were analyzed at baseline, 3, 6, and 9 months. For 3 separate days in a week, including 2 weekdays and 1 weekend day considered typical, each participant recorded all foods and beverages consumed; both type and amount. During group meetings, participants were trained to read food labels and estimate portion sizes in order for amounts to be determined. Upon collection, a trained staff member reviewed each participant's diet record for accuracy and gave suggestions to better comply with the diet if needed. At each data collection period, diet records were entered into the Nutrition Data System for Research (NDSR) (version 4.05_33) by a trained staff member for nutrient composition and energy intake analysis.

### Physical activity

Physical activity (PA) was considered any planned activity of at least moderate-intensity, such as brisk walking, involving major muscle groups that lasted for 10 min or more. Participants were issued pedometers (Accusplit^®^, San Jose, CA) and instructed in their use. Weekly totals for PA in min and steps were reported at each group meeting. Physical activity was initiated after the second clinic meeting and was progressive beginning with 15 min per day, three times/week and reached 50–60 min, 5–6 times/week at month 6. The overall goal was for participants to reach a PA level of 300 min/week at 6 months and maintain that level for the remainder of the study. The progression was intentionally slow to decrease the likelihood of injury as many participants were unaccustomed to regular physical activity.

### Blood pressure

Blood pressure was assessed at baseline, 3, 6, and 9 months. Blood measure was measured on the right arm using a mercury sphygmomanometer with participants lying in the supine position for 5 min prior to measurement [10]. A minimum of two blood pressure measurements were taken. If the first two readings differed by more than 5 mmHg, an additional reading was obtained. The lowest systolic and diastolic blood pressure values were used for analysis [11].

### Adverse events

At each clinic meeting, participants reported any adverse events experienced during the previous week. A form was provided to assess potential adverse events that included questions about nausea, fatigue, flatulence, bad breath, constipation, bloating, stomach cramps, diarrhea, hair loss, change in sleeping patterns, over the counter drugs, insomnia, irritability, body odor, etc. The form containing the list of potential adverse events also included a space to allow participants to explain the adverse event or to describe an event not listed on the form (i.e. "other").

### Statistical analysis

The statistical software package PC-SAS (version 8.2, SAS Institute, Inc., Cary, NC) was employed for all statistical analyses. The level of significance was set at 0.05 for all statistical tests. Descriptive statistics (mean, standard deviation, etc.) were reported for all dependent measures such as body weight, energy and macronutrient intake, etc. The primary outcome was a comparison of body weight during weight maintenance (4 months to 9 months) for the two treatment conditions. For differences in body weight between groups, we applied intention to treat principles by including participants in the analysis who had withdrawn from the study. However, as this was a per protocol study and as there was no difference in statistics when analyzing using intention to treat analysis or per protocol, data and statistics presented hereafter will be reported exclusively for participants who completed the entire duration of the study and all laboratory assessments. T-tests and repeated measures ANOVA were used to detect differences in the change in body weight over time. In addition, mixed effects models were used in order to assess if there was a significant interaction (group*time) for each dependent variable. An autoregressive [AR(1)] covariance structure was assumed for the mixed effects models. In the absence of a significant interaction term, analysis was completed for the main effects of group and time.

## Results

### Participants

A total of 102 participants met the inclusion criteria and initiated the study. The participants were healthy adults (26 men and 76 women), middle-aged, and obese. Ninety-four percent (96/102) of participants were Caucasian, 3% (3/102) were African-American, and 3% (3/102) were Hispanic. Twenty percent (20/102) of participants reported using medications including: anti-hypertensives, diuretics, thyroid medications, or anti-depressants. At baseline, 52 participants were assigned to the low carbohydrate diet group and 50 participants were assigned to the low fat diet group. There were no statistical differences between the low carbohydrate and low fat group at baseline for age, weight, or BMI.

### Attrition and adherence

The low carbohydrate group had 44% attrition and the low fat group had 48% attrition. A summary of reasons for participant attrition and the number of dropouts are included in Table 1. Attrition was greatest during months 4 to 6 for both groups. A total of 55 participants (29 low carbohydrate; 26 low fat) completed all testing and clinic measures at 9 months. There was no statistical difference at baseline for body weight between those who completed the study and those that did not (*P *= 0.14). Characteristics of completers at baseline are presented in Table 2.

**Table 1:** Reasons for withdrawal from the study at each 3 month interval for the low carbohydrate and low fat groups.

	**Base to 3 months**	**3 to 6 months**	**6 to 9 months**	**Total**
**Lack of Attendance**	0/0	2/6	7/1	**9/7**
**Disliked the Dietary Protocol During Maintenance**	NA	3/0	1/1	**4/1**
**Unable to Comply with a Liquid VLED During Weight Loss**	4/3	NA	NA	**4/3**
**Injury**	0/0	2/0	0/0	**2/0**
**Disliked Record Keeping or Other Components of the Program**	0/0	1/4	0/2	**1/6**
**Monetary Conflict**	0/0	1/0	0/1	**1/1**
**Pregnant**	0/0	1/0	0/0	**1/0**
**Work Conflicts**	0/0	1/2	0/1	**1/3**
**Family Conflict**	0/0	0/0	0/2	**0/2**
**Moved from Area**	0/0	0/1	0/0	**0/1**
**Total Dropouts**	4/3	11/13	8/8	**23/24**

Low carbohydrate/low fat

**Table 2:** Baseline characteristics of completers by group.

	**LC**	**LF**	*P*
**N**	29	26	
**Weight (kg)**	109.6 ± 17.3	105.5 ± 15.9	0.36
**BMI (kg/m**^2^**)**	39.1 ± 5.0	37.6 ± 4.9	0.27
**Waist (cm)**	110.5 ± 12.7	106.6 ± 9.6	0.21
**Age (y)**	47.9 ± 10.1	45.7 ± 10.6	0.28

Mean ± SD. LC = Low carbohydrate group. LF = Low fat group.

During the weight loss portion of the study, participants achieved the required number of liquid meal replacements averaging 35 ± 3 per week for the low carbohydrate group and 36 ± 4 per week for the low fat group. Throughout the duration of weight maintenance (months 4–9) participants kept track of daily carbohydrate or fat grams with the low carbohydrate group self-reporting a consumption of 78 ± 30 g of carbohydrate per day and the low fat group self-reporting 39 ± 18 g of fat per day. In addition, analysis of 3-day food records showed the low carbohydrate group consumed an average of 91 ± 39 g of carbohydrate per day equaling ~25% of total kJ from carbohydrate and the low fat group consumed an average of 48 ± 20 g of fat per day averaging ~26% of total kJ from fat, throughout the duration of weight maintenance.

### Pre-study diet

Prior to participation (baseline), energy intake was higher in the low fat group compared with the low carbohydrate group (9439 ± 2428 kJ vs. 7761 ± 1980 kJ; *P *= 0.01). In addition, the low fat group consumed a significantly greater number of grams of carbohydrate, fat, and alcohol (*P *< 0.05); however, the percentage of total energy from carbohydrate, protein, and fat was not significantly different between dietary groups (*P *> 0.05) (Table 3).

**Table 3:** Between group comparisons for macronutrient intake at baseline and across weight maintenance.

		**Weight Maintenance**		
	**Baseline**	**4-Months**	**6-Months**	**9-Months**
**LC Group (n = 29)**				
**Energy Intake (kJ)**	7761 ± 1980*	5180 ± 1630	6318 ± 1819	6259 ± 1611**
**Carbs (g)**	193 ± 52*	75 ± 26	98 ± 48	100 ± 36**
**Protein (g)**	85 ± 28	88 ± 29	99 ± 37	92 ± 24**
**Fat (g)**	83 ± 29*	67 ± 59	81 ± 28	82 ± 34**
**Alcohol (g)**	3 ± 6*	2 ± 4	3 ± 7	2 ± 7**
**Dietary Fiber (g)**	16 ± 5	13 ± 6	14 ± 6	14 ± 6**
**LF Group (n= 26)**				
**Energy Intake (kJ)**	9439 ± 2428	6565 ± 1623	6996 ± 2193	7167 ± 2058
**Carbs (g)**	230 ± 80	215 ± 61	215 ± 72	235 ± 69
**Protein (g)**	90 ± 22	83 ± 23	85 ± 25	83 ± 21
**Fat (g)**	103 ± 32	44 ± 18	50 ± 22	49 ± 21
**Alcohol (g)**	11 ± 15	6 ± 13	11 ± 19	8 ± 16
**Dietary Fiber (g)**	15 ± 5	25 ± 8	26 ± 13	25 ± 8

LC = Low Carbohydrate Group; LF = Low Fat Group. Mean ± SD. Significance set at (P < 0.05).

*Indicates a significant difference from the low fat group at baseline.

**Indicates a significant difference from the low fat group across weight maintenance.

### Weight loss period

During the weight loss period (months 1–3) both groups lost significant amounts of body weight on the VLED. The low carbohydrate group decreased body weight by 20.4 ± 6.2 kg (19%) and the low fat group 19.1 ± 5.4 kg (18%); the difference between groups was not statistically significant. Likewise, BMI, waist circumference, and blood pressure decreased significantly for both groups but differences between groups were not significant.

### Weight maintenance period

**Figure 1:**
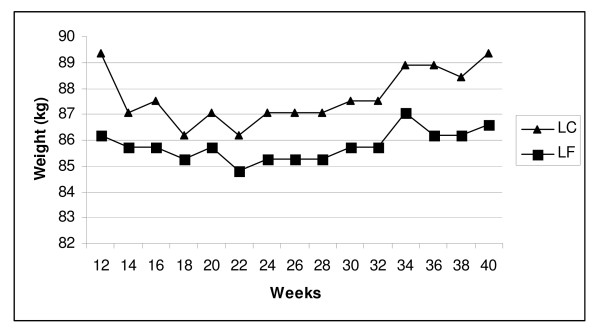
Body weight across weight maintenance for low carbohydrate and low fat groups. LC = low carbohydrate group. LF = low fat group. No significant group*time interaction or within group differences (*P *> 0.05).

**Figure 2:**
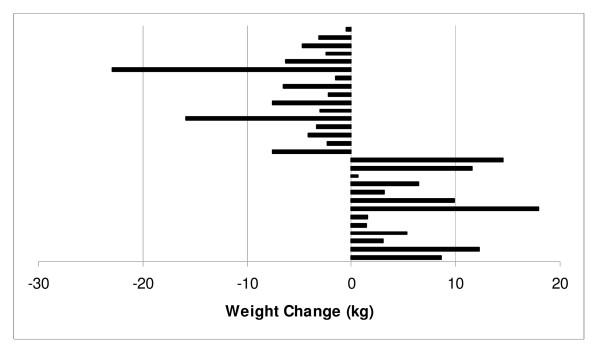
Individual Participant Responses in Body Weight for the Low Carbohydrate Group during Weight Maintenance.

**Figure 3:**
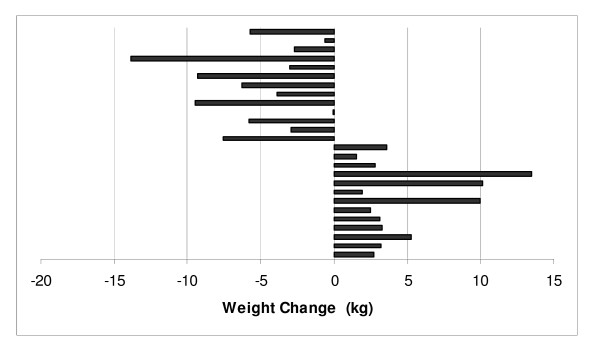
Individual Participant Responses in Body Weight for the Low Fat Group during Weight Maintenance.

Differences in body weight between the two groups were not significant across the 6 months of weight maintenance (*P *= 0.87). Adjusting for medication use and body weight at the beginning of weight maintenance did not influence the outcome. Figure 1 shows body weight at 2 week intervals across the 6 months of weight maintenance. At the beginning of weight maintenance the low carbohydrate group had a body weight of 89.2 ± 14.4 kg that increased to 89.3 ± 16.1 kg at 9 months (*P *= 0.84) and the low fat group had a body weight of 86.3 ± 12.0 kg at 3 months that decreased to 86.0 ± 14.0 kg at 9 months (*P *= 0.96). In the low carbohydrate group, 55% (16/29) of participants decreased their body weight during weight maintenance and 50% (13/26) of participants in the low fat group decreased their body weight during weight maintenance (Figures 2 &3).

Participants in both treatment groups showed a similar response for blood pressure and anthropometrics during the weight maintenance period. At the beginning of weight maintenance, systolic and diastolic blood pressure were not significantly different between groups although systolic blood pressure was slightly higher in the low carbohydrate group (122 vs. 116 mmHg; *P *= 0.08). Over the duration of weight maintenance the low carbohydrate group showed a decrease in systolic blood pressure from 122 ± 11 to 120 ± 10 mmHg and diastolic blood pressure from 75 ± 7 to 73 ± 10 mmHg. The low fat group decreased systolic blood pressure from 116 ± 13 to 111 ± 13 mmHg and diastolic blood pressure from 73 ± 8 to 70 ± 9 mmHg. There were no significant differences in blood pressure between or within groups across the duration of weight maintenance. Likewise, waist circumference and BMI were not statistically different between or within groups at any time period (Table 4).

**Table 4:** Body weight, BMI, and waist circumference for the low carbohydrate and low fat groups during weight maintenance.

	**3-Months**		**6-Months**		**9-Months**	
	LC	LF	LC	LF	LC	LF

**N**	29	26	29	26	29	26
**Body Weight (kg)**	89.2 ± 14.4	86.3 ± 11.9	87.1 ± 15.0	85.3 ± 13.2	89.3 ± 16.1	86.0 ± 14.0
**BMI (kg/m**^2^**)**	31.8 ± 4.5	30.9 ± 4.2	31.1 ± 4.8	30.5 ± 4.5	31.9 ± 5.3	30.8 ± 5.2
**Waist (cm)**	95.9 ± 10.7	91.4 ± 8.7	94.9 ± 14.5	91.4 ± 10.3	96.3 ± 12.7	93.4 ± 10.5

Mean ± SD. LC = low carbohydrate group. LF = low fat group. No significant interactions, no differences at 3 months, or within/between groups differences across the duration of the study (*P *> 0.05).

Comparison of energy and macronutrient intake data during weight maintenance showed that the low carbohydrate group consumed significantly more grams of protein, fat, and percentage of total energy intake from protein and fat compared to the low fat group. The low fat group consumed significantly more total energy, grams of carbohydrate, fiber, and alcohol and a greater percentage of total energy intake from carbohydrate and alcohol compared to the low carbohydrate group. After adjusting for baseline, total energy intake and protein intake were no longer significantly different between groups during weight maintenance (Table 3).

### Physical activity

There was not a significant group*time interaction for min of physical activity during weight maintenance. Physical activity for the low carbohydrate group averaged 268 ± 17 min/week and for the low fat group was 265 ± 23 min/week during weight maintenance. Likewise, there was not a significant group*time interaction for pedometer steps during weight maintenance. The low carbohydrate group averaged approximately 63,000 ± 3200 steps per week and the low fat group 68,000 ± 3500 steps per week during weight maintenance.

### Adverse events

The most commonly reported adverse events in the low carbohydrate group during weight maintenance included headache, constipation, flatus, hair loss, change in sleeping patterns, and stomach cramps. The most commonly reported adverse events in the low fat group during weight maintenance included headache, nausea, fatigue, and diarrhea.

Unexpected adverse events reported in the low carbohydrate diet included dizziness (N = 2), leg cramps (N = 2), a missed menstrual period (N = 1), dandruff (N = 1), decreased sex drive (N = 1), and a 100 mg/dL increase in total cholesterol in 1 participant. Unexpected adverse events reported in low fat diet included change in taste (N = 1) and dizziness (N = 1).

## Discussion

Prevention of weight re-gain is difficult for many individuals [12-14]. The main finding of this investigation was that subsequent to substantial weight loss on a VLED, a low carbohydrate diet and a low fat diet, combined with a clinical weight management program, were similar and effective to prevent weight re-gain over 6 months. For the low carbohydrate group, body weight remained approximately 19% below baseline body weight and the low fat group remained approximately 18% below baseline body weight.

Although both diets were similar to prevent weight re-gain, not all participants responded uniformly to either intervention. It was not surprising that some participants from both dietary groups regained weight after VLED as this has been reported elsewhere [15]. Further, it is well-known that not all individuals that lose weight are successful for weight maintenance [1,2,16]. However, both dietary groups showed similar variation in weight change during the weight maintenance period. Fifty-five percent of participants in the low carbohydrate and 50% of participants in the low fat group continued to decrease their body weight during weight maintenance while the remainder re-gained a portion of their body weight (Figures 2 &3).

To attempt to explain the variability in weight change within each group, we examined energy and macronutrient intake differences between weight gainers and losers and found they were generally not significant; however, a couple of trends are interesting. For the low carbohydrate group, there was no difference in energy intake between the weight gainers and losers but the weight losers averaged 13 g of carbohydrate/day less than the weight gainers (*P *= 0.16). For the low fat group, weight losers consumed 178 kJ/day less (*P *= 0.09) and 7 g of fat/day less (*P *= 0.12) than the weight gainers. These trends imply that the level of carbohydrate or fat restriction for each dietary group may be important for subsequent weight change. Accordingly, the carbohydrate or fat level consumed by the weight gainers may not have been sufficient to maintain energy balance or produce an energy deficit. This is especially likely for the low carbohydrate gainers as the average consumption of carbohydrates was higher than reported in other studies [3,4,6,7].

One statistically significant difference between the low carbohydrate weight gainers and losers is noteworthy. The low carbohydrate weight losers consumed an average of 15 grams of protein/day more than the low carbohydrate weight gainers (*P *= 0.02). This is consistent with human and rodent studies that report that increasing protein intake may be beneficial for weight loss and prevention of weight re-gain [17,18]. For instance, Westerterp-Plantenga et al reported that additional protein intake (18% vs. 15%) resulted in less weight re-gain after 4 weeks of weight loss on a VLED[17]. In addition, increasing the ratio of protein to carbohydrate, as reported by Layman et al, may also be important for continued weight loss and maintenance [19]. Thus, we cannot rule out the possibility that the increased protein intake for the low carbohydrate group weight losers contributed to their continued decrease in body weight. Regardless of the reason for the variability in weight change within each dietary group, it is likely that both diets, if appropriately applied and adhered to, will yield a measure of success for weight maintenance in some individuals. Perhaps one of the most interesting questions arising from this study for future investigations is "how to determine or predict which individuals are most likely to succeed consuming a specific diet".

For the present study, attrition was similar for both groups. The primary reason for attrition during weight maintenance for both groups was lack of attendance at group meetings. Participants would not always provide reasons for their unwillingness to continue attendance or they intentionally discontinued correspondence and were removed from the study after dropping below the required attendance level (75%). As a result, we are left to conjecture as to why some participants discontinued attendance. We recognize that the high attrition in both groups is unfortunate and represents a weakness of the study. However, other similar studies have reported high rates of attrition up to 38% for low carbohydrate groups and 46% for low fat groups [4,6,7]. In a recent meta-analysis of 5 low carbohydrate vs. low fat trials reported by Nordmann et al, attrition rates were 30% and 43% for a low carbohydrate and low fat diet, respectively, after 6 months and 38% and 46%, respectively, after 12 months [20]. As mentioned earlier, in attempt to limit attrition bias, we included an intent to treat analysis which did not change the statistical significance for any variable.

Physical activity is an important component of successful weight maintenance [21,22]. Interventions that promote lifestyle changes along with PA have shown better weight maintenance than interventions that do not have these components [21]. For the present study, PA was an important component and was similar for both groups. Both groups were prescribed an identical amount of PA and there was little variation. As a result, any difference in body weight change between groups during weight maintenance is not likely due to differences in PA.

Some participants were on prescription medications during the study. However, medication use was generally stable and statistical adjustment for medication use did not significantly influence body weight outcomes. As a result, we do not believe that medication use was a confounding factor. Further, the results are likely to generalize fairly well to an overweight or obese adult population, who are typically taking 1 or more medications such as blood pressure, depression, and lipids [23].

There were more total adverse events in the low carbohydrate group than in the low fat group. However, it should be noted that when adverse events were considered excluding the re-feeding period (month 4) the total number of adverse events reported were essentially the same for both groups. It is possible that the transition from a liquid VLED to solid food is more difficult when consuming a low carbohydrate diet or that our method of re-feeding can be improved to smooth this transition. Commonly reported adverse events for the low carbohydrate group were consistent with other studies, specifically, constipation, and diarrhea [7,24]. One participant in the low carbohydrate group had an unexplainable increase in total cholesterol after 3 months on the low carbohydrate diet (total cholesterol increased from 136 to 306 mg/dL). This participant was advised to seek medical attention immediately.

We recognize that there are several limitations with this study. 1) The diet and PA data were self-reported. There are known biases and limitations with self-reported data, such as under-reporting energy intake [25]. Nevertheless, diet records are commonly used and acceptable research instruments. 2) There were considerably more women than men and so results were not reported by gender. 3) We chose to use a quazi-experimental design with the site being assigned as either low carbohydrate of low fat rather than to randomize individual participants to a particular group. This was done because the popularity of the Atkins diet was at its height during our data collection. We felt that if participants assigned to different dietary protocols were in the same group or location there would be increased likelihood of data contamination by participants choosing to follow the dietary protocol of their choice rather than their assignment. Further, we did not use a cluster design and analyzed the data by individual participants. Had we analyzed the data by clinic assignment, the sample size would have been n = 2, insufficient for a cluster, and would likely have biased the results. Nevertheless, we recognize that the study design can be improved for future studies by randomization of participants or randomization by clinic using a cluster design of sufficient sample size. 5) The data collected for adverse events may be biased due to the assessment method. Adverse event data was collected from participants by administering a single sided page that listed specific adverse events seen in other low carbohydrate and low fat studies. We may have inadvertently prompted the participants to consider a specific adverse event they would not necessarily have reported had it not been listed.

## Conclusion

This study addressed a significant gap in the current literature by comparing body weight in participants on either a low carbohydrate diet or low fat diet during a 6 month weight maintenance period following weight loss. The primary finding of this study was that a low carbohydrate and low fat diet, combined with a clinical weight management program, are comparable for body weight maintenance over 6 months; however, there was significant variation in weight change within each group.

## Abbreviations

BW = Body Weight (Abstract Only)

PA = Physical Activity

g = grams

min = minutes

kJ = kilojoules

kg = kilograms

BMI = body mass index

VLED = very low-energy diet

REE = resting energy expenditure

cm = centimeters

## Competing interests

Dr. Mary C. Vernon receives honoraria as a consultant for Mrs. Veronica Atkins, Chairperson of the Board of Directors for the Robert C. Atkins Foundation. The Atkins Foundation provided partial support for this project. The remaining authors declare that they have no competing interests.

## Authors' contributions

All authors approved the final manuscript. JD conceived the study design with input from JL, RW, DS, CG, SH, MV, and EW. JL, CC, and ES coordinated and executed all aspects of the study with input from RW, DS, CG, and SH. MV provided medical oversight of all subjects. Data were analyzed by JL with support by SH. The manuscript was prepared by JL and JD, and all authors contributed to the editing of the final manuscript.
